# Dynamics of Microbial Communities and Antibiotic Resistance Genes During *Arthrospira platensis* Cultivation in Swine Wastewater

**DOI:** 10.3390/biology15171484

**Published:** 2026-09-01

**Authors:** Authen Promariya, Sekbunkorn Treenarat, Chanchanok Duangsri, Niyada Lansubsakul, Sorawit Powtongsook, Wanthanee Khetkorn, Wuttinun Raksajit

**Affiliations:** 1 Program of Animal Health Technology, Faculty of Veterinary Technology, Kasetsart University, Bangkok 10900, Thailand; authen.pr@ku.th (A.P.); sekbunkorn.tr@ku.th (S.T.); 2 Department of Animal Science, Faculty of Agricultural Technology and Agro-Industry, Rajamangala University of Technology Suvarnabhumi, Phranakhon Si Ayutthaya 13000, Thailand; chanchanok.d@rmutsb.ac.th; 3 Department of Physiology, Faculty of Veterinary Medicine, Kasetsart University, Bangkok 10900, Thailand; niyada.la@ku.th; 4 Center of Excellence for Marine Biotechnology, Department of Marine Science, Faculty of Science, Chulalongkorn University, Bangkok 10330, Thailand; sorawit@biotec.or.th; 5 National Center for Genetic Engineering and Biotechnology (BIOTEC), Pathum Thani 12120, Thailand; 6 Division of Biology, Faculty of Science and Technology, Rajamangala University of Technology Thanyaburi (RMUTT), Pathum Thani 12110, Thailand; wanthanee_k@rmutt.ac.th

**Keywords:** antibiotic resistance genes (ARGs), *Arthrospira platensis*, microbial community dynamics, nutrient removal, photobioreactor, swine wastewater

## Abstract

Swine wastewater is a nutrient-rich waste stream with considerable potential for cyanobacterial cultivation and resource recovery. However, its impacts on microbial community dynamics and antibiotic resistance genes (ARGs) remain poorly understood. This study evaluated the cultivation of *Arthrospira platensis* in a medium containing 25% swine wastewater and 75% Zarrouk medium (SWW_25_) using a flat-panel photobioreactor. *A. platensis* achieved a biomass concentration of 1.58 ± 0.02 g/L, a biomass productivity of 0.11 ± 0.01 g/L/d, and a specific growth rate of 0.26 ± 0.01 d^−1^. Simultaneously, the system removed 98.5% of ammonium nitrogen and 80% of orthophosphate. Metagenomic analysis indicated changes in bacterial community composition during *A. platensis* cultivation. Microbial community analysis revealed shifts in relative abundance after cultivation, likely associated with changes in community composition following *Arthrospira* biomass enrichment, while Proteobacteria, Firmicutes, and Synergistota remained among the dominant phyla. In addition, ARG profiling indicated a decline in ARG diversity and read counts during cultivation, whereas *tet(X)* remained the only detectable ARG after *A. platensis* cultivation. Collectively, these findings suggest that *A. platensis* cultivation under non-axenic conditions was associated with biomass production, nutrient removal, and changes in microbial community composition and ARG profiles in swine wastewater.

## Simple Summary

Swine wastewater contains high levels of nutrients that can cause environmental pollution if improperly managed. This study evaluated the potential of *Arthrospira platensis* for swine wastewater utilization and biomass production, with particular emphasis on microbial community dynamics and antibiotic resistance genes (ARGs). *A. platensis* grew in a wastewater-based medium and was associated with nutrient removal and shifts in microbial community composition under the tested conditions. ARG profiling further revealed reductions in ARG diversity and read counts during cultivation. Overall, these findings demonstrate the potential of *A. platensis* cultivation as a resource-recovery approach for swine wastewater utilization, while providing insights into associated microbial community and ARG dynamics.

## 1. Introduction

The swine industry is a major component of Thailand’s agricultural sector [1]. In 2025, Thailand produced approximately 1.736 million tons of pork, supporting domestic consumption and exports, with quality assurance implemented from the farm level [2]. Globally, increasing demand for pork has driven continuous expansion of pig production, which currently exceeds one billion animals per year [3]. As production intensifies, the volume of swine wastewater (SWW) generated from manure, urine, wash water, and feed residues also increases. Pig farming is estimated to account for approximately 76.8% of livestock wastewater discharges, making it a major source of agricultural pollution [4]. SWW is typically characterized by high organic matter content, elevated chemical oxygen demand (COD), and high concentrations of ammonium (NH_4_^+^), orthophosphate (PO_4_^3−^), and other contaminants [5]. When released without appropriate treatment, these contaminants can impair water quality through eutrophication and oxygen depletion, while also contributing to greenhouse gas emissions [6], and the dissemination of antimicrobial residues [7]. Conventional aerobic wastewater treatment systems are effective for reducing organic matter and nutrient loads. However, they are generally energy-intensive and primarily designed for pollutant removal rather than resource recovery [8]. Therefore, circular bioeconomy-based approaches have gained increasing attention as alternative strategies to convert wastewater-derived nutrients into valuable biomass. In this context, nutrient-rich SWW represents a potential cultivation medium for cyanobacteria, enabling simultaneous nutrient removal and biomass production [9].

During cultivation, inorganic nutrients are assimilated into biomass, thereby reducing nutrient concentrations while generating valuable bioproducts. In addition to wastewater treatment, cyanobacterial cultivation offers opportunities for carbon capture, nutrient recycling, and water reuse. However, large-scale implementation remains constrained by the cost of synthetic cultivation media. The utilization of nutrient-rich waste streams, including agricultural and industrial wastewaters, has therefore gained attention as an alternative approach to reduce reliance on conventional media while supporting integrated wastewater treatment and biomass production [10]. The resulting biomass can subsequently be valorized into a range of value-added products, such as biofuels, bioplastics, pigments and other high-value biochemicals [11,12].

Among cyanobacteria, *Arthrospira platensis* (recently reclassified as *Limnospira platensis*), commonly known as Spirulina, has attracted considerable attention due to its rapid growth, high photosynthetic efficiency, and ability to accumulate commercially valuable metabolites. The biomass of *A. platensis* is rich in proteins, carbohydrates, essential fatty acids, vitamins, and bioactive pigments, including chlorophyll-*a*, carotenoids, and phycocyanin, contributing to its widespread cultivation and commercial applications [12]. Importantly, the biochemical composition of *A. platensis* can be modulated by cultivation conditions and nutrient availability. For example, phosphorus limitation has been reported to enhance carbohydrate accumulation [13], whereas nitrogen-rich conditions promote protein synthesis and biomass productivity [14]. These characteristics enable the production of biomass with tailored biochemical profiles while supporting nutrient removal from wastewater-based cultivation systems. Due to its metabolic flexibility and ability to utilize inorganic nutrients, *A. platensis* is considered a promising candidate for cultivation using nutrient-rich waste streams as alternatives to conventional synthetic media. Wastewater containing nitrogen, phosphorus, and trace elements can provide essential nutrients for biomass production while reducing cultivation costs [15]. Accordingly, numerous studies have investigated the application of *A. platensis* for treating various wastewater streams, including brewery effluents [16], dairy farm wastewater [17], aquaculture wastewater [18], and winery wastewater [19]. In these systems, wastewater has been applied either directly or after pretreatment and dilution to reduce potential inhibitory effects and improve cultivation performance [20].

Despite the growing interest in using *A. platensis* for wastewater treatment, its application in SWW remains insufficiently understood, particularly regarding the integration of nutrient removal, biomass production, microbial community dynamics, and antibiotic resistance gene (ARG) profiles. SWW contains complex microbial communities, residual antibiotics, and ARGs that may influence treatment performance and environmental safety. Cyanobacteria–bacteria interactions have been reported to contribute to nutrient cycling, organic matter transformation, and biomass productivity through complementary metabolic processes [21,22]. However, their potential roles in shaping microbial community succession and ARG dynamics during *A. platensis*-based SWW cultivation remain largely unexplored. This knowledge gap limits the optimization and environmental evaluation of cyanobacteria-based SWW treatment systems.

Therefore, this study aimed to (i) evaluate the growth performance and nutrient removal efficiency of *A. platensis* cultivated in swine wastewater, (ii) characterize changes in bacterial community composition, and (iii) assess the diversity and read counts of antibiotic resistance genes (ARGs) during cultivation. By integrating biomass production, nutrient removal, and metagenomic analyses, this study provides insight into microbial and ARG dynamics associated with *A. platensis*-based wastewater utilization and contributes to a better understanding of cyanobacterial cultivation in swine wastewater.

## 2. Materials and Methods

### 2.1. Water Sampling and Quality Analysis

Swine wastewater (SWW) treated through a biogas system was randomly collected from a local swine farm in Nakhon Pathom Province, Thailand, in May 2025. Samples were taken using glass bottles with screw caps and immediately transported to the laboratory for analysis. Composite sampling was conducted by collecting wastewater from the surface at a depth of approximately 1–2 feet at three locations: the center, left edge, and right edge of the retention pond. Three subsamples of 500 mL each were collected and combined to form a representative composite sample. The composite sample was then filtered through a mesh filter to remove suspended solids and stored at −20 °C until further analysis. Prior to analysis, SWW samples were filtered through a 0.45 µm membrane filter. The filtrates were then analyzed for ammonium nitrogen (NH_4_^+^–N), nitrate nitrogen (NO_3_^−^–N), nitrite nitrogen (NO_2_^−^–N), orthophosphate (PO_4_^3−^–P), soluble chemical oxygen demand (sCOD), total dissolved solids (TDS), calcium (Ca), magnesium (Mg), and pH according to the Standard Methods for the Examination of Water and Wastewater (APHA/AWWA/WEF) [23]. All analyses were performed by Central Laboratory (Bangkok, Thailand) Co., Ltd.

### 2.2. Microorganism and Optimization of Culture Conditions in a Photobioreactor

**Figure 1:**
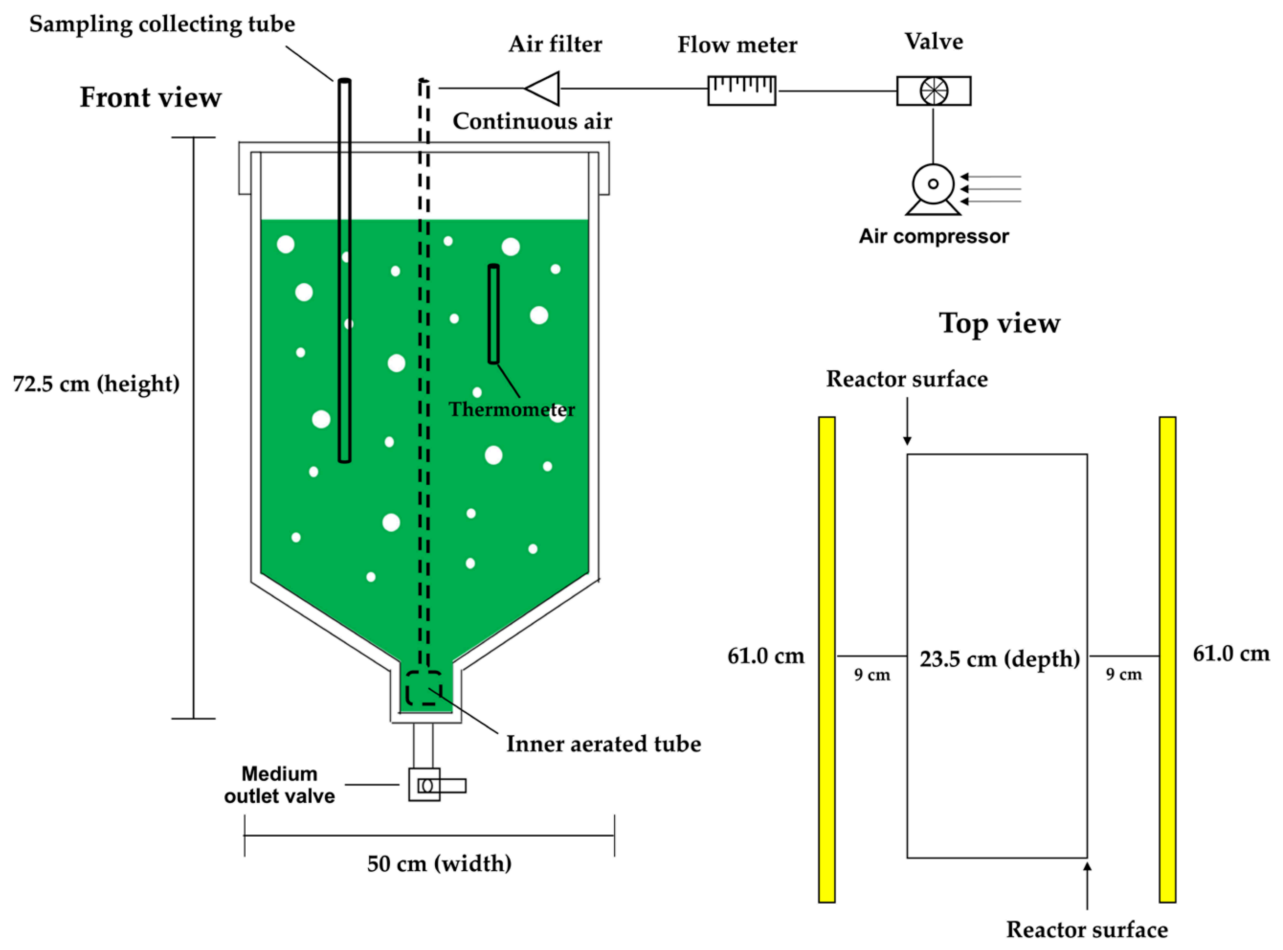
Schematic representation of the 50 L flat-panel photobioreactor (FPP) employed for the cultivation of *A. platensis*.

The cyanobacterium *Arthrospira platensis* IFRPD1182 was obtained from the Institute of Food Research and Product Development (IFRPD). Flask-scale cultivation was initially performed in 250 mL Erlenmeyer flasks containing 50 mL of cultivation medium consisting of Zarrouk medium or swine wastewater (SWW)-supplemented Zarrouk medium. Five medium formulations were prepared: control (50 mL Zarrouk medium), SWW_25_ (37.5 mL Zarrouk medium + 12.5 mL SWW), SWW_50_ (25 mL Zarrouk medium + 25 mL SWW), SWW_75_ (12.5 mL Zarrouk medium + 37.5 mL SWW), and SWW_100_ (50 mL SWW). The initial pH of all media was adjusted to 10.0 ± 0.5 using 5 N NaOH. Cultures were incubated on a rotary shaker at 120 rpm under continuous LED illumination (40 μmol photons/m^2^/s) at 32 ± 2 °C for 14 d. Flask-scale experiments were performed using three independent biological replicates, with triplicate measurements obtained for each biological replicate. After cultivation, *A. platensis* biomass was harvested by filtration and used as the inoculum for scale-up cultivation in a flat-panel photobioreactor (FPP) at an initial biomass concentration of approximately 0.1 g/L. The acrylic FPP had a working volume of approximately 50 L and consisted of an acrylic culture chamber equipped with an air filter and an internal aeration tube positioned at the bottom to provide continuous aeration and mixing. The system was also equipped with a medium outlet valve and a sampling collection tube for culture sampling, while a thermometer was used to monitor the culture temperature (Figure 1). The FPP measured 23.5 cm (depth) × 50.0 cm (width) × 72.5 cm (height). SWW_25_ was prepared by mixing 25% (*v*/*v*) swine wastewater (SWW) with 75% (*v*/*v*) Zarrouk medium and introduced into the reactor to the working volume. Continuous mixing and aeration were provided through the bottom sparger at an aeration rate of 0.1 vvm. The reactor was illuminated by four 40-W white LED lamps (4000 lm per lamp; 61.0 cm in length), with two lamps positioned on each side of the reactor at a distance of 9 cm from the reactor surface, resulting in an incident light intensity of approximately 300 μmol photons/m^2^/s. The FPP was operated under controlled cultivation conditions, with the temperature and pH maintained at 32 ± 2 °C and 10.0 ± 0.5, respectively, throughout the 14-d cultivation period. An uninoculated SWW_25_ control was operated under the same reactor configuration and cultivation conditions, including medium composition, illumination, aeration, temperature, pH, and cultivation duration, but without inoculation with *A. platensis*. For the FPP experiments, three independent cultivation runs were conducted, and each run was considered one biological replicate.

### 2.3. Growth Performance, Biomass Production, and Pigment Analysis

Growth was monitored by measuring the optical density at 730 nm (OD_730_) using a Genesys 30 spectrophotometer (Thermo Fisher Scientific, Waltham, MA, USA). Because wastewater constituents may interfere with optical measurements, the corresponding uninoculated wastewater-based medium was used as the blank for background correction. Blank-corrected OD_730_ values were used to evaluate *A. platensis* growth. Biomass concentration was determined independently by the dry weight method. Cells were harvested by filtration, dried to a constant weight, and weighed. The biomass concentration was expressed as g/L. Biomass productivity was subsequently calculated according to Equation (1):

(1) $$\mathrm{Biomass}\;\mathrm{productivity}\;(g/L/d)\;=\;\frac{\left(x_{2}-x_{1}\right)}{\left(t_{2}-t_{1}\right)}$$

where x_1_ and x_2_ represent biomass concentrations (g/L) at the beginning and end of the cultivation period, respectively, and t represents the cultivation time (d).

The average specific growth rate (μ) was calculated during the exponential phase of the growth curve [24] as the natural logarithm of the ratio of biomass concentration at the end of the exponential phase (x_f_) to the initial biomass concentration (x_i_), divided by the corresponding cultivation time, as shown in Equation (2):

(2) $$\mu (d^{-1})=\frac{\left(ln\;x_{f}-ln\;x_{i}\right)}{\left(t_{f}-t_{i}\right)}$$

where x_f_ and x_i_ represent the biomass concentrations at times t_f_ and t_i_, respectively.

The doubling time (T_d_) was calculated based on Equation (3):

(3) $$T_{d}\left(d\right)=\frac{0.693}{µ}$$

where μ is the specific growth rate.

Chlorophyll *a* (Chl-*a*) and carotenoids (Car) were quantified using the absorption coefficient method. Briefly, methanolic extracts of centrifuged culture samples were analyzed at 665, 470, and 720 nm using the same spectrophotometer [25]. Furthermore, phycocyanin (PC) was determined from phosphate buffer extracts after ultrasonic disruption and centrifugation, and absorbance was measured at 620 and 652 nm using the same spectrophotometer [26].

### 2.4. Determination of Nutrient Remaining (%) and Removal Efficiency (%)

Ammonium nitrogen (NH_4_^+^–N) remaining (%) can be calculated using Equation (4):

(4) $${{NH}_{4}}^{+}-N\;remaining\left(\%\right)=\frac{N_{\left(f\right)}}{N_{\left(i\right)}}\times 100$$

where N_(i)_ is the initial NH_4_^+^–N concentration and N_(f)_ is the final NH_4_^+^–N concentration.

Orthophosphate (PO_4_^3−^–P) remaining (%) can be calculated using Equation (5):

(5) $${{PO}_{4}}^{3-}-P\;remaining\left(\%\right)=\frac{P_{\left(f\right)}}{P_{\left(i\right)}}\times 100$$

where P_(i)_ is the initial PO_4_^3−^–P concentration and P_(f)_ is the final PO_4_^3^–P concentration.

The removal efficiencies of NH_4_^+^–N and PO_4_^3−^–P were calculated from their respective remaining percentages using the following Equation (6):

Nutrient removal efficiency (%) = 100 − Nutrient remaining (%)(6)

### 2.5. Metagenomics Sequencing and Data Analysis

The ZymoBIOMICS^®^ Shotgun Metagenomic Sequencing Service (Zymo Research, Irvine, CA, USA) was used to characterize microbial community composition and antimicrobial resistance gene (ARG) profiles. Culture samples were collected from three independent cultivation runs for each experimental condition, filtered through 0.2 μm membrane filters, and genomic DNA was extracted using the ZymoBIOMICS^®^-96 MagBead DNA Kit (Zymo Research, Irvine, CA, USA) according to the manufacturer’s instructions. For each experimental condition, genomic DNA from three independent cultivation runs was pooled prior to library preparation. Thus, one pooled DNA sample and one shotgun metagenomic sequencing library were generated per condition. Libraries were prepared using the Nextera^®^ DNA Flex Library Prep Kit (Illumina, San Diego, CA, USA) with up to 100 ng of input DNA and internal single-index 8-bp barcodes (Nextera^®^ and TruSeq^®^). Library quality and concentration were assessed using a TapeStation^®^ system (Agilent Technologies, Santa Clara, CA, USA), followed by library quantification by qPCR and sequencing on an Illumina HiSeq^®^ platform. No de novo assembly was performed. Instead, downstream taxonomic and ARG analyses were conducted directly from quality-filtered sequencing reads. Raw reads were quality-filtered and adapter-trimmed using Trimmomatic (v0.33) [27] with a 6-bp sliding window and a minimum quality score of 20, and reads shorter than 70 bp were discarded. Microbial community composition was profiled using Sourmash (v4.1.0) [28] against the GTDB (R07-RS207) and GenBank (v2022.03) reference databases for taxonomic identification of bacteria, archaea, viruses, protozoa, and fungi. The resulting taxonomic profiles were analyzed using QIIME 2 [29] to assess alpha and beta diversity and relative abundances at the phylum, order, and genus levels. ARGs were identified directly from the quality-filtered reads using the DIAMOND sequence aligner (v0.4.7) [30] against the NCBI AMR Database (v2020-08-09). ARG assignments were retained using minimum sequence identity and alignment coverage thresholds of 80% and 90%, respectively. ARG profiles were generated from the detected resistance genes and reported as read counts for each gene. Because ARGs were identified directly from sequencing reads without assembly, the detected ARGs were not assigned to specific bacterial hosts.

### 2.6. Statistical Analysis

Statistical analysis was performed using one-way analysis of variance (ANOVA), followed by Tukey’s honestly significant difference (HSD) test for multiple comparisons. Differences were considered statistically significant at *p* < 0.05. Statistical analyses were performed using SPSS version 22 (IBM Corp., Armonk, NY, USA).

## 3. Results

### 3.1. Characteristics of Swine Wastewater (SWW)

The raw SWW was characterized by high nutrient and pollutant loads, including elevated concentrations of NH_4_^+^–N, PO_4_^3−^–P, Ca, Mg, sCOD, and TDS (Table 1), indicating substantial levels of nitrogen, phosphorus, organic matter, and dissolved solids. Supplementation of swine wastewater with Zarrouk medium (SWW_25_) markedly altered the wastewater composition, resulting in decreases in most measured parameters, an increase in NO_3_^−^–N, and no detectable NO_2_^−^–N. The increase in NO_3_^−^–N concentration after preparation of SWW_25_ was attributable to the nitrate supplied by the Zarrouk medium. During the 14-day cultivation period with *A. platensis*, NH_4_^+^–N, NO_3_^−^–N, PO_4_^3−^–P, Ca, Mg, and sCOD concentrations showed a continuous decline, whereas NO_2_^−^–N was not detected. Moreover, pH increased from approximately 7.5 in the raw wastewater to around 10.0 following supplementation with Zarrouk medium and remained alkaline throughout cultivation. Although TDS decreased substantially following preparation of the SWW_25_ medium, it was not determined further during *A. platensis* cultivation.

### 3.2. Growth and Pigment Production of A. platensis Cultivated in SWW

**Figure 2:**
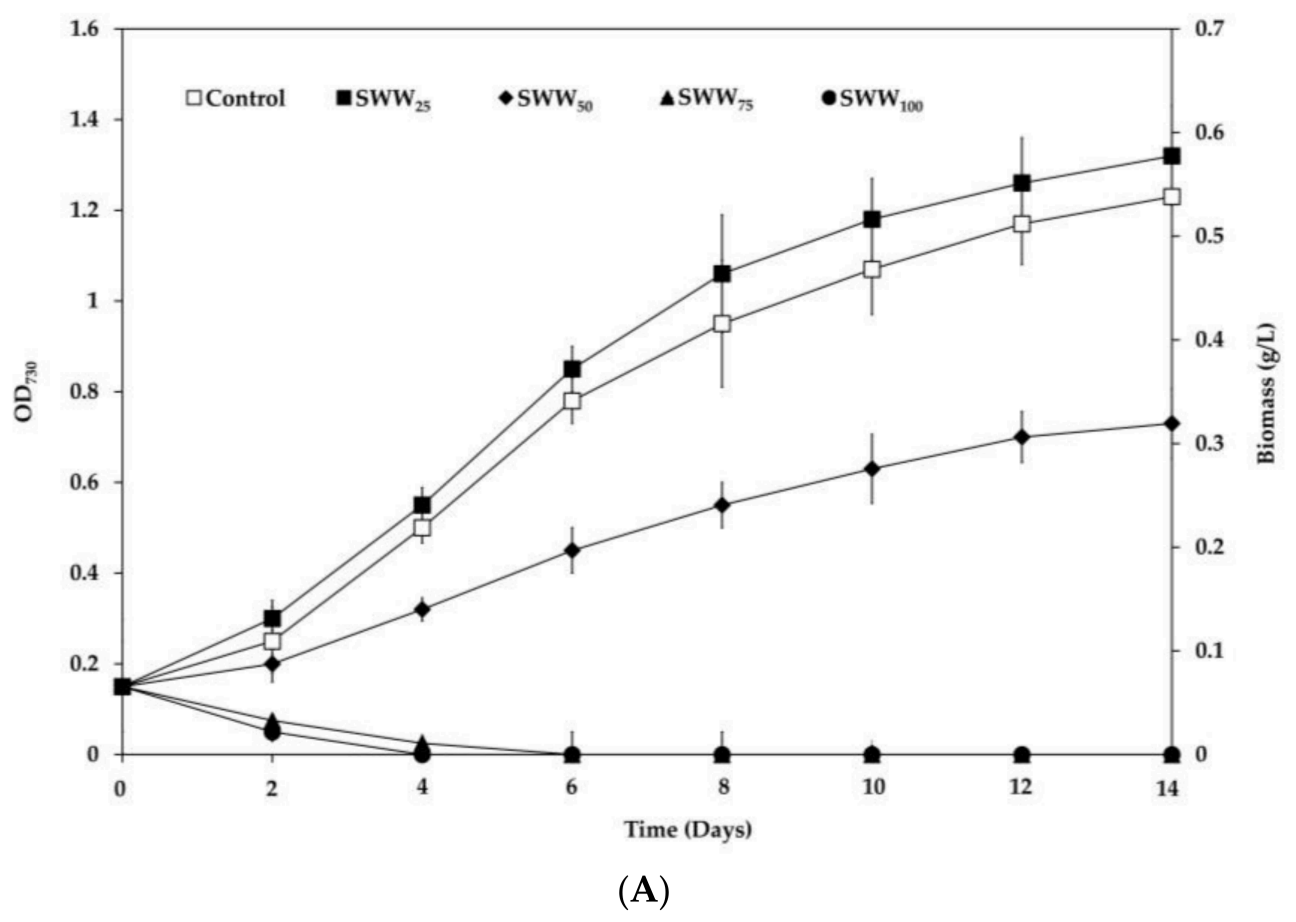

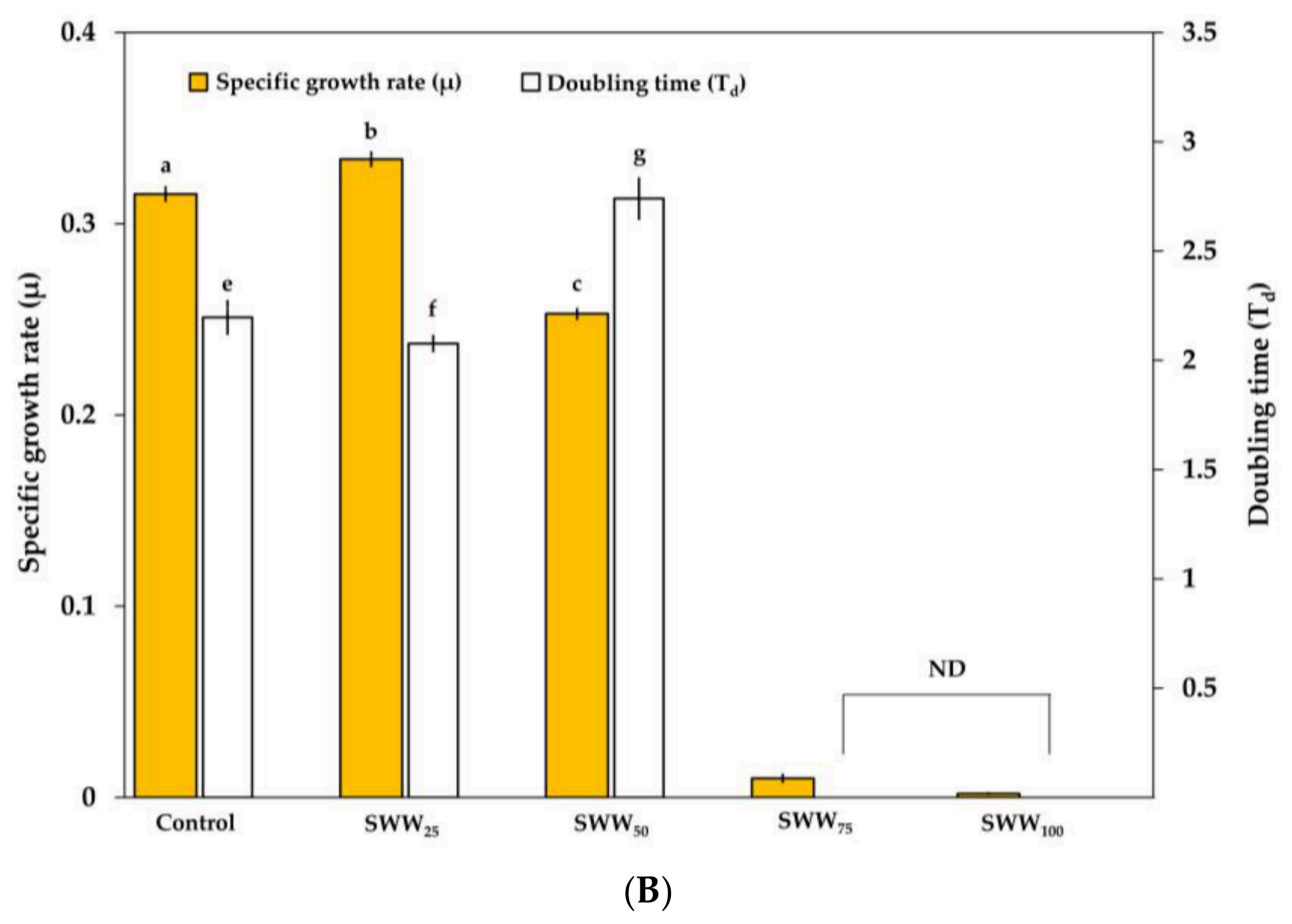
Growth of *A. platensis* cultivated in Zarrouk’s medium (control, ☐) and swine wastewater (SWW)-supplemented Zarrouk medium (SWW_25_ (■), SWW_50_ (◆), SWW_75_ (▲), and SWW_100_ (●)) during 14 days of cultivation. (**A**) Optical density at 730 nm (OD_730_; primary *y*-axis) and biomass concentration (g/L; secondary *y*-axis), determined independently using the dry weight method. (**B**) Specific growth rate (μ, d^−1^; dark-yellow bars; primary *y*-axis) and doubling time (T_d_, d; white bars; secondary *y*-axis). Different lowercase letters above the bars indicate significant differences among treatments according to Tukey’s HSD test (*p* < 0.05). ND, not determined.

Figure 2 shows that *A. platensis* was cultivated in Zarrouk’s medium (control) and subsequently exposed to increasing concentrations of SWW (SWW_25_, SWW_50_, SWW_75_, and SWW_100_). As shown in Figure 2A, the OD_730_ pattern in SWW_25_ closely resembled that of the control over 14 d, yielding final biomass concentrations of 0.54 ± 0.05 and 0.58 ± 0.04 g/L, respectively. In contrast, growth in SWW_50_ was reduced, with a lower biomass concentration of 0.32 ± 0.05 g/L on day 14. Furthermore, higher SWW concentrations (SWW_75_ and SWW_100_) markedly inhibited growth, showing slower initial growth within the first 2 d and complete cessation of proliferation after day 4. Consistent with these growth patterns, the specific growth rate (μ) in SWW_25_ was 0.33 ± 0.01 d^−1^. The corresponding doubling time (T_d_) was 2.07 ± 0.01 d. Both parameters were comparable to those of the control (Figure 2B). In SWW_50_, μ decreased to 0.25 ± 0.01 d^−1^, whereas T_d_ increased to 2.74 ± 0.01 d compared with the control (Figure 2B).

**Figure 3:**
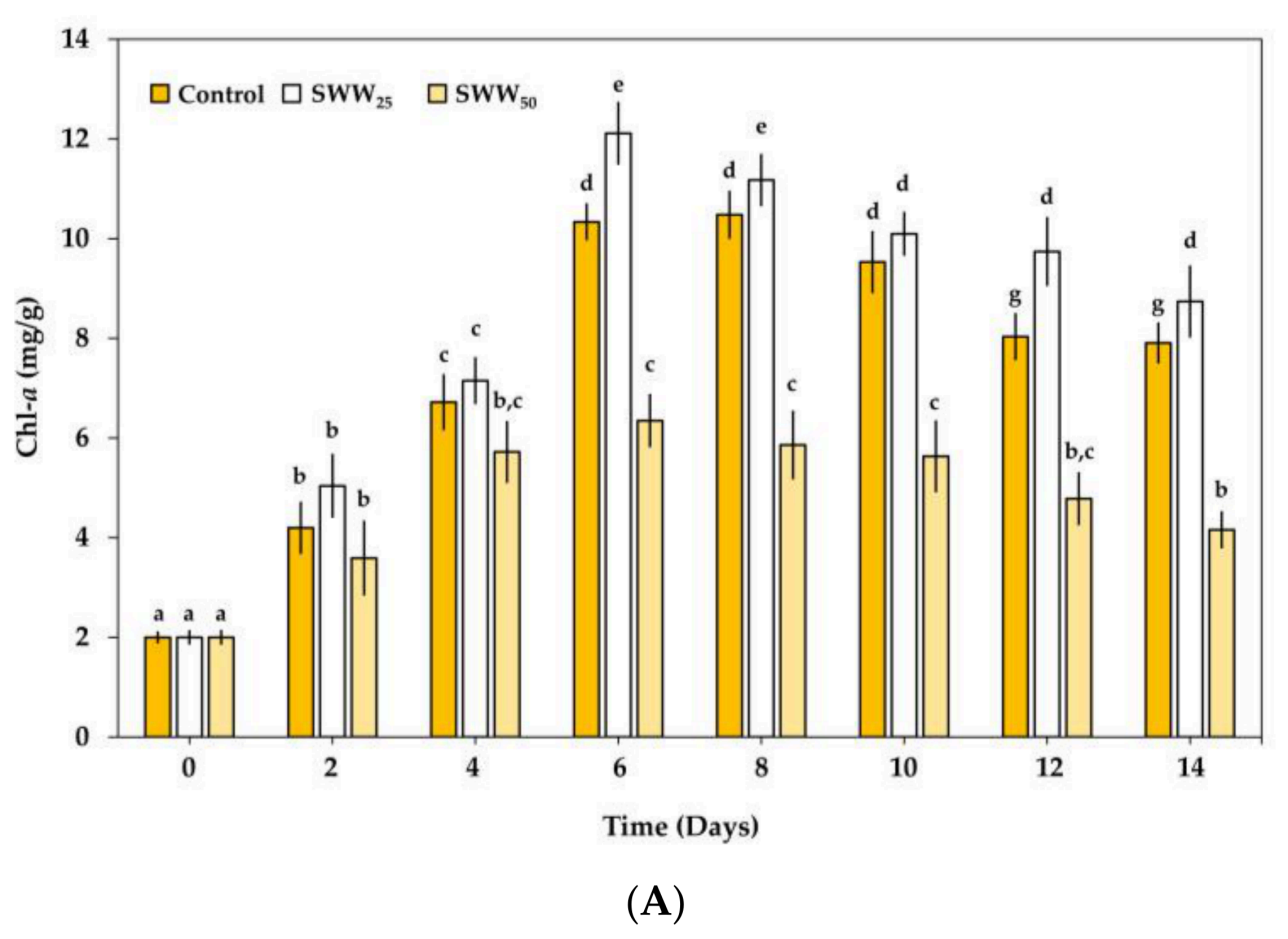

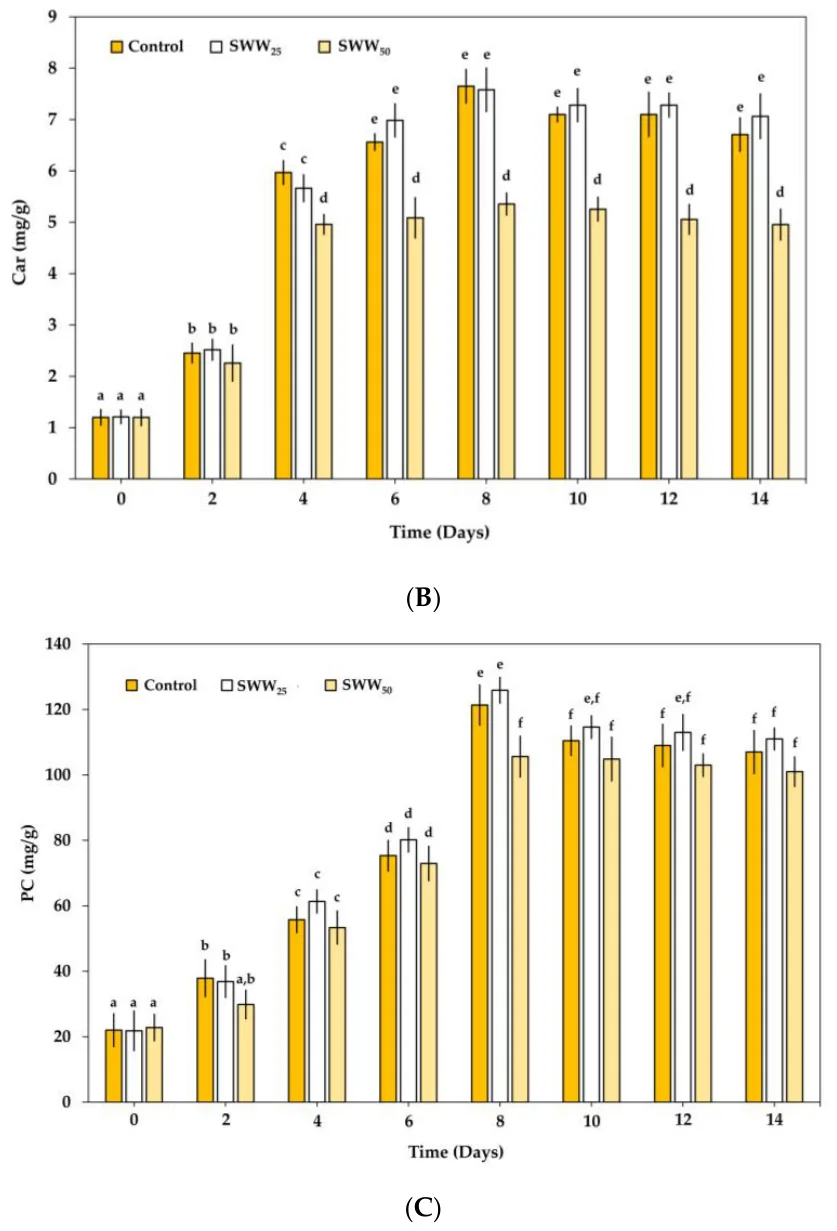
Pigment contents of *A. platensis* cultivated in Zarrouk medium (control, dark-yellow bars), SWW_25_ (white bars), and SWW_50_ (light-yellow bars) over 14 days. (**A**) chlorophyll-*a* (Chl *a*), (**B**) total carotenoids (Car), and (**C**) phycocyanin (PC). Different lowercase letters above the bars indicate significant differences among treatments according to Tukey’s HSD test (*p* < 0.05).

Similarly, pigment production was influenced by SWW concentration. Pigment accumulation in *A. platensis* cultivated in SWW_25_ followed a trend similar to the control over the 14-d period (Figure 3). Notably, by day 6 and 8, the highest Chl-*a*, Car, and PC contents were observed in SWW_25_, reaching 12.11 ± 0.18, 7.58 ± 0.15, and 125.85 ± 0.01 mg/g, respectively. In contrast, cultivation in SWW_50_ resulted in lower pigment levels, with Chl-*a*, Car, and PC contents of 6.43 ± 0.16, 5.14 ± 0.13, and 105.62 ± 0.02 mg/g, respectively (Figure 3A–C).

As shown in Table 2, cultivation in the FPP using SWW_25_ resulted in a biomass concentration of 1.58 ± 0.02 g/L and biomass productivity of 0.11 ± 0.01 g/L/d, representing increases of 7.5% and 10.0%, respectively, compared with the control. In contrast, μ decreased by 10.3% to 0.26 ± 0.01 d^−1^, while T_d_ increased by 11.8% to 2.66 ± 0.01 d. Pigment contents were also higher in the SWW_25_ treatment, with Chl-*a*, Car, and PC reaching 27.74 ± 4.71, 15.54 ± 6.63, and 113.50 ± 5.88 mg/g, respectively, corresponding to increases of 8.9%, 15.2%, and 2.5% compared with the control.

### 3.3. Changes in NH_4_^+^–N and PO_4_^3−^–P During Cultivation in the Photobioreactor System

**Figure 4:**
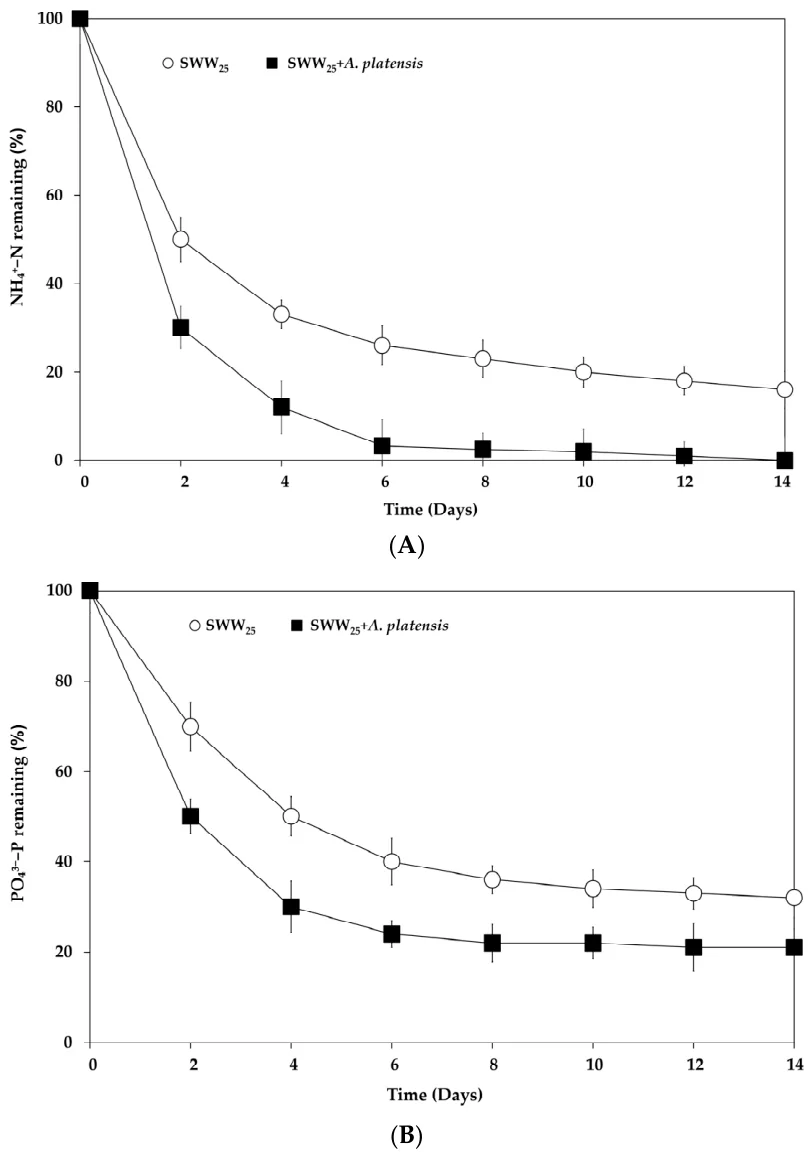
Percentage of (**A**) NH_4_^+^–N and (**B**) PO_4_^3−^–P remaining during the cultivation of *A. platensis* in SWW_25_ over 14 d in a 50 L flat-panel photobioreactor (FPP). Open circle (○) represent uninoculated SWW_25_, and filled squares (■) represent SWW_25_ inoculated with *A. platensis*.

Figure 4 shows the percentage of NH_4_^+^–N and PO_4_^3−^–P remaining during the cultivation of *A. platensis* in SWW_25_ in the 50 L FPP. In the inoculated reactor, the percentage of NH_4_^+^–N remaining decreased rapidly during the first 6 d and continued to decline throughout the cultivation period (Figure 4A), reaching approximately 1.5% (0.63 ± 0.02 mg/L) of the initial NH_4_^+^–N concentration (42.23 ± 1.92 mg/L) after 14 d. The uninoculated control also exhibited a reduction in NH_4_^+^–N, although the decrease was less pronounced, with approximately 18% of the initial NH_4_^+^–N remaining after 14 d. A similar trend was observed for PO_4_^3−^–P (Figure 4B). The percentage of PO_4_^3−^–P remaining decreased progressively throughout the cultivation period, reaching approximately 20.0% (28.31 ± 0.53 mg/L) of the initial concentration (141.56 ± 1.32 mg/L) after 14 d in the inoculated reactor. In contrast, the uninoculated control retained approximately 31% of the initial PO_4_^3−^–P after 14 d. These results correspond to NH_4_^+^–N and PO_4_^3−^–P removal efficiencies of approximately 98.5% and 80.0%, respectively, in the inoculated reactor.

### 3.4. Microbial Community Composition in SWW Samples

**Figure 5:**
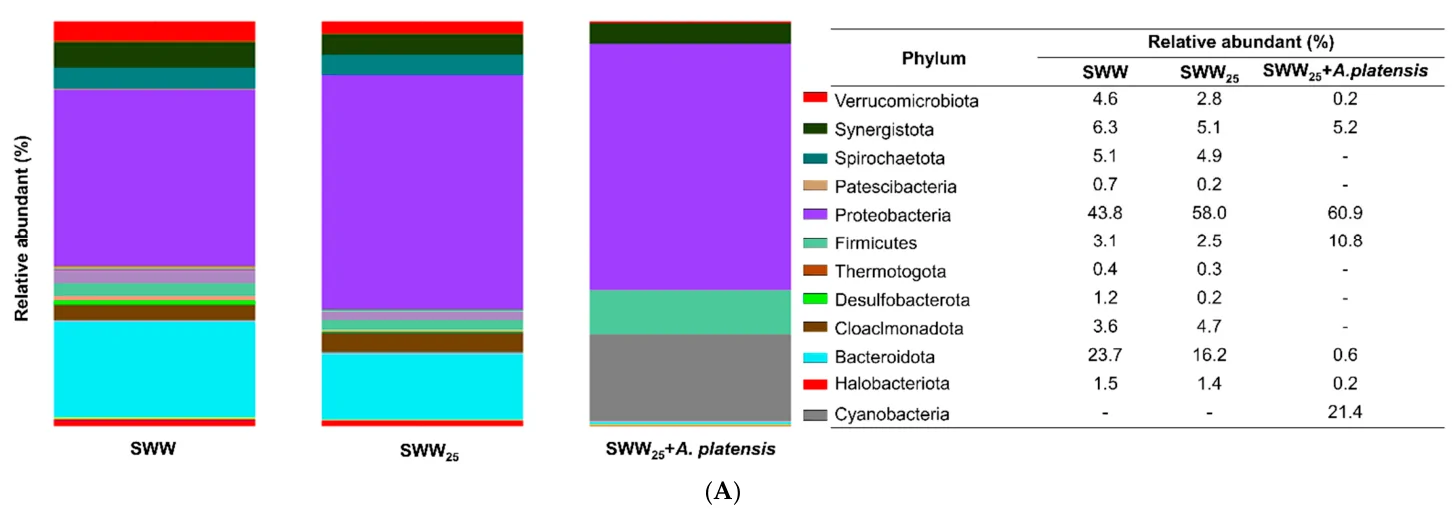

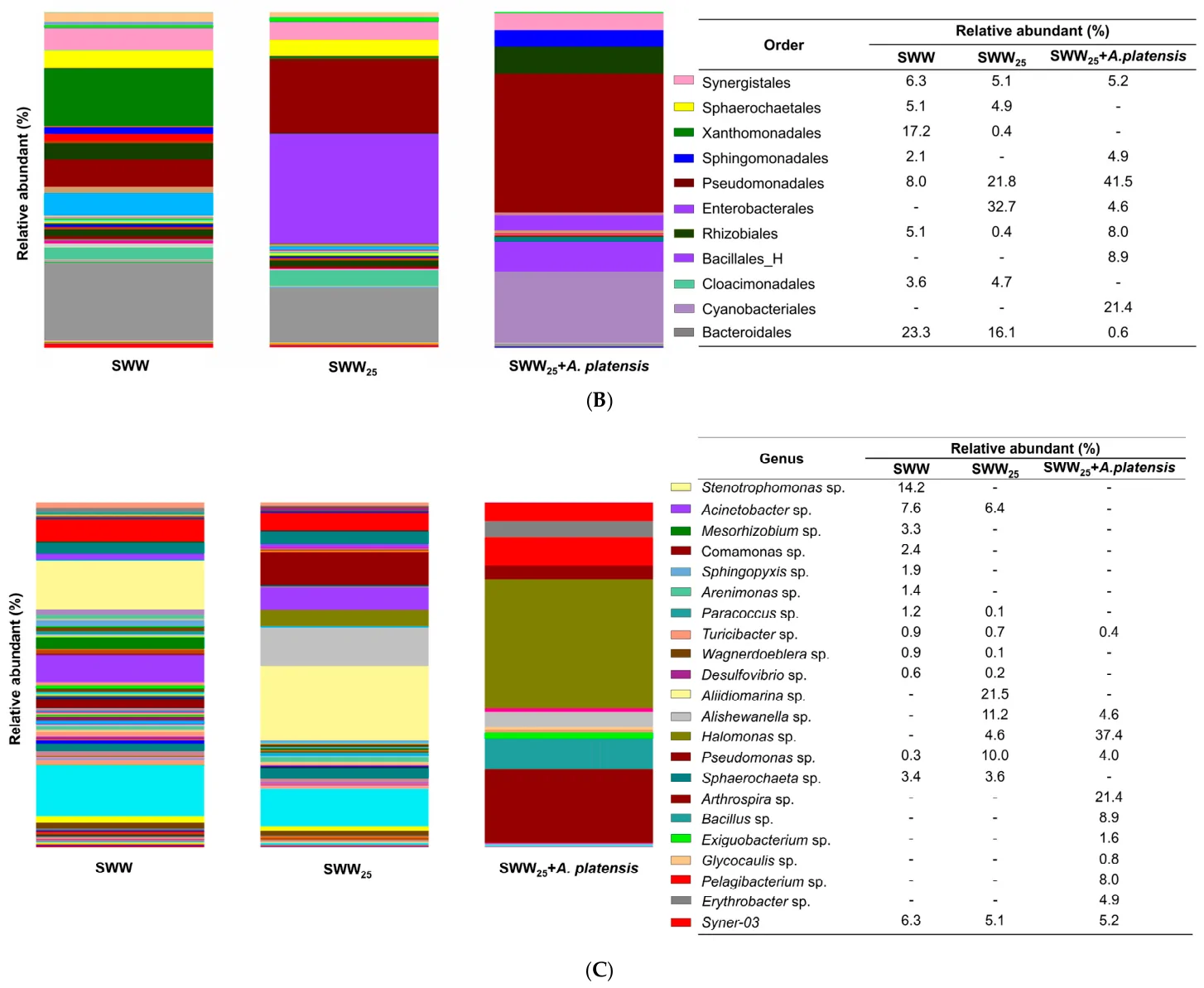
Relative abundance profiles of bacterial communities at the (**A**) phylum, (**B**) order, and (**C**) genus levels in SWW, SWW_25_, and SWW_25_ inoculated with *A. platensis*, based on shotgun metagenomic sequencing.

The taxa described below are representative examples of the observed changes in relative community composition and are not intended to represent all taxa detected in the metagenomic analysis. The comparison of microbial communities at the phylum level is presented in Figure 5A. Initially, SWW_25_ was dominated by eleven bacterial phyla, including Proteobacteria (58%), Bacteroidota (16.2%), Synergistota (5.1%), Spirochaetota (4.9%), Cloacimonadota (4.7%), Verrucomicrobiota (2.8%), Firmicutes (2.5%), Halobacteriota (1.4%), Thermotogota (0.3%), Desulfobacterota (0.2%), and Patescibacteria (0.2%). Following the cultivation of *A. platensis*, five phyla (Spirochaetota, Cloacimonadota, Desulfobacterota, Thermotogota, and Patescibacteria) were not detected above the applied analytical threshold. The relative abundances of Bacteroidota, Verrucomicrobiota and Halobacteriota also decreased in the post-cultivation community profile. In contrast, Proteobacteria, Cyanobacteria, Firmicutes, and Synergistota became the predominant phyla, accounting for 60.9%, 21.4%, 10.8%, and 5.2% of the community, respectively.

At the order level, Enterobacterales was the dominant group in SWW_25_, representing 32.7% of the total community, followed by Pseudomonadales (21.8%), Bacteroidales (16.1%), Synergistales (5.1%), Sphaerochaetales (4.9%), Cloacimonadales (4.7%), Xanthomonadales (0.4%), and Rhizobiales (0.4%) (Figure 5B). Following the cultivation of *A. platensis*, Pseudomonadales remained the most abundant order (41.5%), whereas Cyanobacteriales (21.4%), Bacillales_H (8.9%), Rhizobiales (8.0%), Synergistales (5.2%), and Sphingomonadales (4.9%) showed increased relative abundances. Similarly, at the genus level, SWW_25_ was characterized by the predominance of *Aliidiomarina* sp. (21.5%), *Alishewanella* sp. (11.2%), *Pseudomonas* sp. (10.0%), *Acinetobacter* sp. (6.4%), *Syner-03* (5.1%), *Halomonas* sp. (4.6%), *Sphaerochaeta* sp. (3.6%), *Turicibater* sp. (0.7%), *Desulfovibrio* sp. (0.2%), *Paracoccus* sp. (0.1%), and *Wagnerdoeblera* sp. (0.1%) (Figure 5C). Following the cultivation of *A. platensis*, several genera were not detected above the applied analytical threshold, while *Turicibacter* sp., *Alishewanella* sp. and *Pseudomonas* sp. showed decreased relative abundances. In contrast, several genera became detectable in the post-cultivation community profile, including *Bacillus* sp. (8.9%), *Pelagibacterium* sp. (8.0%), *Syner-03* (5.2%), *Erythrobacter* sp. (4.9%), *Exiguobacterium* sp. (1.6%), and *Glycocaulis* sp. (0.8%). The relative abundance of *Halomonas* sp. also increased to 37.4%.

### 3.5. Antibiotic Resistance Gene Profiles in SWW Samples

**Figure 6:**
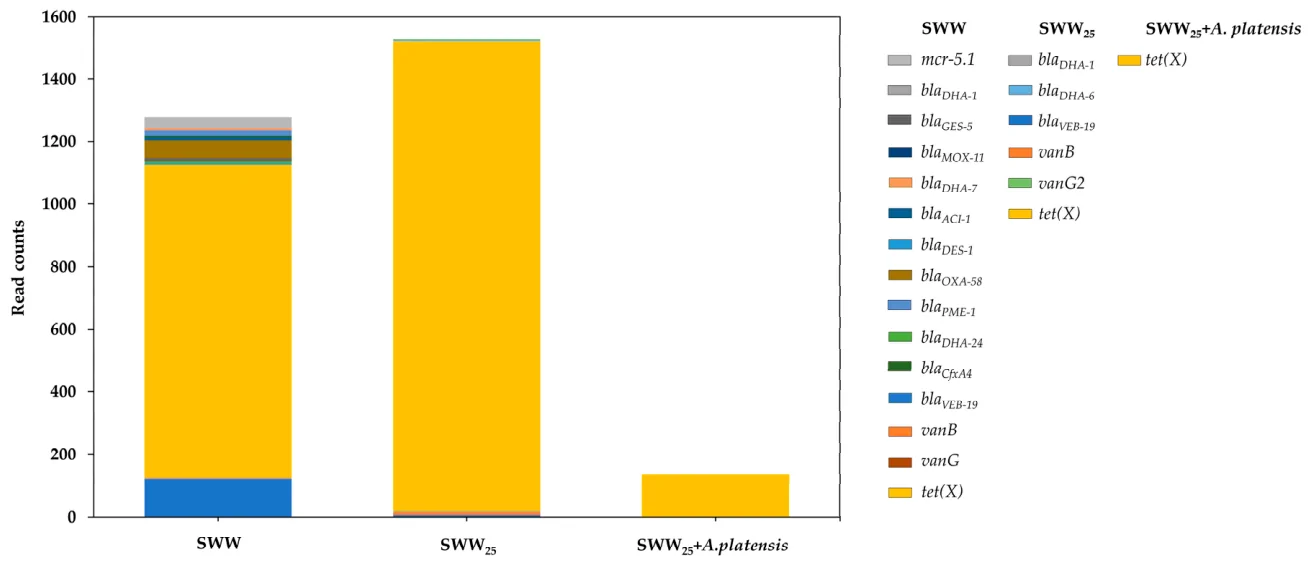
Read counts of antibiotic resistance genes (ARGs) detected in SWW, SWW_25_, and SWW_25_ inoculated with *A. platensis*.

Antibiotic resistance gene (ARG) profiling revealed distinct ARG profiles among SWW, SWW_25_, and SWW_25_ inoculated with *A. platensis* (Figure 6). SWW exhibited the highest ARG diversity and read counts. Among the detected ARG classes, β-lactam resistance genes were the most frequently detected, with 11 genes identified, including *bla_VEB-19_*, *bla_GES-5_*, *bla_PME-1_*, *bla_CfxA4_*, *bla_ACI-1_*, *bla_DHA-1_*, *bla_DHA-7_*, *bla_DHA-24_*, *bla_DES-1_*, *bla_MOX-11_*, and *bla_OXA-58_.* In addition, polymyxin resistance was represented by *mcr-5.1*, glycopeptide resistance genes (*vanB* and *vanG*) and tetracycline resistance was primarily represented by *tet(X)*. Following preparation of the SWW_25_ medium, both ARG diversity and ARG read counts decreased. Only a limited number of ARGs, including *bla_DHA-6_*, *bla_DHA-1_*, *vanB*, and *vanG2*, remained detectable with relatively low read counts. In contrast, *tet(X)* remained detectable and represented a larger propotion of the remaining ARG reads, although its read counts were also low. Following *A. platensis* cultivation, ARG diversity and read counts decreased further, with *tet(X)* remaining the only detected ARG. Overall, the detected ARGs profiles showed reduced diversity and read abundance from SWW to SWW_25_ and after *A. platensis* cultivation.

## 4. Discussion

The cyanobacterium *Arthrospira* (formerly known as *Spirulina*) can assimilate a wide range of inorganic nutrients, including ammonium (NH_4_^+^–N), nitrate (NO_3_^−^–N), nitrite (NO_2_^−^–N), and orthophosphate (PO_4_^3−^–P), thereby contributing to nutrient removal during wastewater cultivation [31]. The wastewater characteristics used in this study are summarized in Table 1. *Arthrospira* preferentially assimilates ammonium (NH_4_^+^–N), which can be directly incorporated into cellular metabolism. However, elevated ammonium concentrations may inhibit cyanobacterial growth, with optimal NH_4_^+^–N concentrations reported at approximately 50 mg/L and growth inhibition occurring at 100–200 mg/L [32]. Although the tolerance threshold varies among cyanobacterial species [33], *A. platensis* has been reported to exhibit greater tolerance to elevated ammonium concentrations under mixotrophic conditions [32]. In the present study, the lower ammonium concentration in SWW_25_ appeared to provide more favorable conditions for *A. platensis* cultivation, whereas the higher concentrations in SWW_75_ and SWW_100_ may have contributed to the limited growth observed under these conditions. NH_4_^+^–N removal likely resulted from a combination of nutrient assimilation by *A. platensis* and the indigenous microbial community, together with physiochemical process. Because cultivation was conducted under non-axenic and alkaline conditions (pH 10.0 ± 0.5) with continuous aeration, the relative contributions of these mechanisms could not be distinguished. Under these conditions, ammonia volatilization may also have contributed to NH_4_^+^–N reduction in addition to biological assimilation [34].

A reduction in sCOD was also observed during cultivation, potentially reflecting organic carbon utilization by *A. platensis* and indigenous microorganisms and interactions within the microbial consortium. However, the respective contributions of cyanobacterial assimilation and bacterial degradation were not determined. Previous studies have reported considerable variation in COD removal by photosynthetic microorganisms cultivated in digested piggery wastewater, likely due to differences in wastewater characteristics, cultivation conditions, and microbial species. For example, *A. platensis* achieved a lower COD removal efficiency (38.4%) than *Scenedesmus obliquus* (>67.3%), *Chlorella pyrenoidosa* (63.3%), and *Oedogonium* sp. (62.5%) after 7 d of cultivation [35]. In the present study, sCOD removal was lower than NH_4_^+^–N removal, suggesting that organic carbon utilization was less pronounced under the conditions employed. Nevertheless, sCOD in SWW_25_ decreased by up to 80% after 14 d of non-axenic *A. platensis* cultivation, comparable to the 84% COD removal reported for *A. maxima* within 20 d [36]. The non-axenic cultivation also implies potential interactions between *A. platensis* and indigenous bacteria throughout the cultivation period. These interactions may have been either competitive or cooperative, as bacterial growth could compete with *A. platensis* for nutrients, inorganic carbon, and light, while microbial interactions may also contribute to organic matter degradation and nutrient cycling. However, bacterial biomass and activity were not quantified separately in the present study. Therefore, the extent and nature of these interactions remain unclear. Further studies are needed to elucidate the respective roles and interactions of *A. platensis* and indigenous bacterial communities during wastewater cultivation.

In addition, orthophosphate (PO_4_^3−^–P) concentration decreased after 14 d of cultivation. This reduction may have resulted from a combination of biological phosphorus assimilation and physiochemical processes. Under the non-axenic conditions, both *A. platensis* and the indigenous microbial community may have contributed to phosphorus assimilation, while the decreases in Ca and Mg concentrations suggest that phosphate precipitation may also have occurred under the alkaline cultivation conditions. Previous studies have shown that phosphorus removal in wastewater systems is closely associated with nitrogen availability, reflecting the balanced nutrient requirements for microbial growth [37]. Comparison with the uninoculated SWW_25_ control maintained under the same alkaline conditions further indicated that *A. platensis* cultivation was associated with additional nutrient changes. Nevertheless, the respective contributions of biological assimilation and physicochemical processes could not be quantified, and the effects of alkaline pH and other abiotic processes cannot be completely excluded [38].

The observed nutrient removal was accompanied by enhanced biomass production in SWW_25_. A fourfold increase in *A. platensis* biomass has been reported after 12 d of cultivation in diluted swine wastewater [39], while 25% swine wastewater increased *A. maxima* cell density approximately twofold over 18 d (μ = 0.16 d^−1^) [36]. Consistent with these findings, SWW_25_ supported biomass production and pigment accumulation in the present study, suggesting that appropriate wastewater dilution may provide sufficient nutrients while reducing the inhibitory effects associated with concentrated swine wastewater [40]. In contrast, higher swine wastewater concentrations (75–100%) generally resulted in poorer cyanobacterial growth [36], consistent with the limited growth observed in SWW_75_ and SWW_100_. This inhibition may be related to increased turbidity and ammonium concentrations, which can restrict light penetration and impair photosynthetic performance, respectively [41]. Beyond biomass production, wastewater-based cultivation may also provide an opportunity for value-added compound production. Previous studies have reported phycocyanin production by *A. maxima* under non-axenic conditions [42] and astaxanthin production by *Haematococcus pluvialis* in sterile wastewater [43]. Similarly, *A. platensis* cultivated in SWW_25_ in the present study accumulated Chl-*a*, Car, and PC, indicating that diluted swine wastewater may serve not only as a nutrient source for biomass production but also as a potential medium for the generation of value-added pigments.

As summarized in Table 3, the performance of cyanobacterial and microalgal cultivation systems varies considerably across studies, owing to differences in wastewater characteristics, cultivation conditions, reactor configuration, and species employed. For example, *Chlorella sorokiniana* AK-1 cultivated in non-axenic 50% swine wastewater achieved nitrogen and phosphorus removal efficiencies of 78.3% and 97.7%, respectively, within 15 d [44], whereas axenic *Chlorella vulgaris* cultivated in sterile digested piggery wastewater removed 72.48% nitrogen and 86.93% phosphorus within 10 d [45]. Pilot-scale cultivation of *Anabaena* sp., *Dolichospermum* sp., and *Spirulina* sp. LEB 18 under non-axenic conditions has likewise demonstrated effective nutrient removal in wastewater treatment systems [46,47]. The effective removal of NH_4_^+^–N and PO_4_^3−^–P observed in SWW_25_ was comparable to values reported for other wastewater-based cyanobacterial cultivation systems. These findings suggest that nutrient-rich SWW-supplemented Zarrouk medium can support cyanobacterial growth and nutrient assimilation under the cultivation conditions evaluated. Nevertheless, the contribution of individual processes, including cyanobacterial uptake, bacterial activity, and physicochemical effects, requires further investigation.

The application of cyanobacteria–bacteria consortia in wastewater treatment has received increasing attention [48,49,50], as complementary metabolic activities and metabolite exchange may enhance nutrient transformation. In the present study, comparison of raw SWW with SWW_25_ showed a marked reduction in microbial diversity following wastewater dilution. Furthermore, comparison of SWW_25_ with and without *A. platensis* revealed additional shifts in microbial community composition. These findings suggest that wastewater dilution influenced the microbial community, while the presence of *A. platensis* may have further contributed to changes in community structure. As shown in Figure 5, Proteobacteria and Bacteroidota remained the dominant phyla in SWW_25_, consistent with previous reports identifying these groups as major members of swine wastewater microbial communities involved in organic matter degradation and nitrogen transformation [51]. Following *A. platensis* cultivation, shifts in relative community composition were observed at the phylum, order, and genus levels. These observations suggest that cultivation under non-axenic conditions was associated with changes in microbial community composition beyond those observed in SWW_25_ alone. However, because these results are based on relative-abundance data, the observed shifts may partly reflect compositional changes associated with the increased abundance of *Arthrospira* rather than absolute changes in individual microbial taxa.

Similar observations have been reported in swine wastewater treatment systems, where metagenomic analyses identified the coexistence of cyanobacteria and diverse microbial communities. These studies also reported associations between cyanobacteria–bacterial consortia and effective nutrient removal, suggesting that microbial interactions may contribute to wastewater treatment performance [52,53]. Nevertheless, the underlying mechanisms remain unclear and require further investigation.

Moreover, ARG analysis indicated that the diversity and read counts of detected ARGs decreased after preparation of the SWW_25_ medium and declined further following *A. platensis* cultivation. Untreated swine wastewater contained a diverse resistome dominated by β-lactam resistance genes, together with polymyxin, glycopeptide, and tetracycline resistance determinants. The predominance of β-lactamase genes, including *bla_GES_*, *bla_DHA_*, and *bla_OXA_* variants, is consistent with previous reports showing that livestock and veterinary hospital-related wastewaters harbor diverse ARGs associated with antibiotic use in animal production and veterinary medicine [54,55,56]. The detection of *mcr-5.1* and glycopeptide resistance genes (*vanB* and *vanG*) further indicates the presence of resistance determinants associated with clinically important antibiotic classes in swine wastewater [57]. After preparation of the SWW_25_ medium, the diversity and read counts of detected ARGs were reduced, and following *A. platensis* cultivation, *tet(X)* remained the only consistently detected ARG. These changes coincided with shifts in microbial community composition, suggesting that alterations in community structure may have influenced the observed ARG profiles [54]. However, the mechanisms underlying these changes remain unclear. The combined effects of SWW_25_ preparation, alkaline conditions, *A. platensis*-associated microbial interactions, and changes in community composition may have contributed to the observed patterns and warrant further investigation.

Throughout the experimental treatments, *tet(X)* remained detectable, indicating its persistence under the conditions evaluated. The *tet(X)* gene encodes an enzyme that inactivates tetracycline antibiotics and has been widely reported in agricultural and environmental settings [58]. Its persistence despite the overall reduction in ARG diversity and read counts suggests that some resistance determinants may be more persistent than others during wastewater-based cultivation. Nevertheless, because ARGs were identified directly from sequencing reads without de novo assembly, these results should be interpreted as changes in detectable ARG read profiles, and specific ARG–host associations could not be confirmed.

## 5. Conclusions

Swine wastewater provided a nutrient-rich substrate for the cultivation of *Arthrospira platensis*, supporting biomass production and nutrient removal. In the SWW_25_-FPP system, *A. platensis* achieved a biomass concentration of 1.58 ± 0.02 g/L while reducing NH_4_^+^–N and PO_4_^3−^–P concentrations by approximately 98.5% and 80%, respectively, within 14 d. Biomass accumulation was accompanied by increased chlorophyll-*a*, carotenoid, and phycocyanin contents, indicating active physiological growth. Metagenomic analysis revealed shifts in bacterial community composition during *A. platensis* cultivation, accompanied by reduced diversity and read counts of detected ARGs. Most ARG classes showed lower read counts during cultivation, whereas *tet(X)* remained detectable at low read counts. These findings indicate that non-axenic *A. platensis* cultivation can support nutrient utilization and biomass production while being associated with shifts in microbial community composition and ARG profiles in swine wastewater. Overall, this study provides insights into microbial community dynamics during cyanobacterial cultivation and highlights the potential of *A. platensis*-based systems for swine wastewater utilization and biomass production, with concurrent changes in detectable ARG profiles.

## Figures and Tables

**Table 1:** Physicochemical characteristics of SWW.

Parameters	SWW		
	Raw SWW	SWW_25_	SWW_25_ + *A. platensis*
NH_4_^+^–N (mg/L)	242.93 ± 4.36	42.23 ± 1.92	0.63 ± 0.02
NO_3_^−^–N (mg/L)	<0.060	452.7 ± 15.2	40.54 ± 10.4
NO_2_^−^–N (mg/L)	<0.010	<0.003	<0.003
PO_4_^3−^–P (mg/L)	161.56 ± 2.43	141.56 ± 1.32	28.31 ± 0.53
Ca (mg/L)	64.91 ± 0.01	15.86 ± 0.01	4.53 ± 0.01
Mg (mg/L)	44.25 ± 0.01	13.52 ± 0.02	3.16 ± 0.02
sCOD (mg/L)	2386.62 ± 56.05	596.43 ± 32.05	119.32 ± 12.15
TDS (mg/L)	8541.65 ± 112.43	2123.41 ± 96.43	ND *
pH	7.51 ± 0.02	10.01 ± 0.50	10.03 ± 0.50

* Not determined.

**Table 2:** Growth performance and pigment contents of *A. platensis* cultivated in the FPP system.

Culture	Growth Performance				Pigments		
	Biomass	Productivity	μ	T_d_	Chl-*a*	Car	PC
	(g/L)	(g/L/d)	(d^−1^)	(d)	(mg/g)	(mg/g)	(mg/g)
Control	1.47 ± 0.02	0.10 ± 0.01	0.29 ± 0.01	2.38 ± 0.01	25.48 ± 6.20	13.49 ± 5.65	110.70 ± 4.38
SWW_25_	1.58 ± 0.02	0.11 ± 0.01	0.26 ± 0.01	2.66 ± 0.01	27.74 ± 4.71	15.54 ± 6.63	113.50 ± 5.88

**Table 3:** Performance of cyanobacteria and microalgae in wastewater treatment and resource recovery.

Wastewater Type	Microalgal Strain	Photobioreactor Configuration	Cultivation Conditions	Products (Yield)	Nutrient Removal (%, Time)	Ref.
Non-sterile piggery wastewater + Zarrouk medium	*Arthrospira maxima*	A raceway pond (600 L)	3 × 2 × 0.3 m^3^, five-blade paddle wheel	Phycocyanin (38.74 mg/mg)	Sulphate (52.28%, 12 d)	[42]
Non-sterile swine wastewater + Zarrouk medium (SWW_25_)	*Arthrospira platensis*	Flat panel (50 L)	300 μmol/m^2^/s, light 24 h, air, 0.1 vvm	Biomass (1.58 g/L), Chl-*a* (27.74 mg/g), Car (15.54 mg/g), PC (113.50 mg/g)	NH_4_^+^–N (98.5%, 14 d), PO_4_^3−^–P (80.0%, 14 d)	This study
Non-sterile pig slurry + culture medium	*Anabaena* sp., *Dolichospermum* sp.	A raceway reactor	1.04 m^3^, 7.13 m^2^, 0.12 m, culture depth, CO_2_	Biomass productivity (20.9–28.0 g/m^2^/d)	NH_4_^+^–N (55.55–75.2%, 7 d)	[46]
Non-sterile swine wastewater + BG11 medium	*Chlorella sorokiniana* AK-1	Glass (1 L)	150 μmol/m^2^/s, 27 °C, 300 rpm, air, 0.1 vvm, 2% CO_2_	Biomass (5.45 g/L)	Total nitrogen (78.3%, 15 d), Total phosphorus (97.7%, 15 d)	[44]
Sterile piggery wastewater + BG11 medium	*Chlorella vulgaris*	Thin-film flat plate (25 L)	150 μmol/m^2^/s, light 12 h: dark 12 h, air, 0.2 vvm	Biomass (0.63 g/L), Chl a + b (18.34 mg/L)	Total nitrogen (72.48%, 10 d), Total phosphorus (86.93%, 10 d)	[45]
Sterile primary-treated wastewater + NIES-C medium	*Haematococcus pluvialis*	High-intensity photoincubator	200 μmol/m^2^/s, light 12 h: dark 12 h, 5% CO_2_	Biomass (1.43 g/L), Astaxanthin (83.9 mg/L)	Nitrate (100%, 7 d), Phosphate (72%, 7 d)	[43]
Non-sterile wastewater from the desalination process	*Spirulina* sp. LEB 18	A semi-continuous raceway	2.2 × 0.9 × 0.35 m^3^, 420 L/h, 24 h agitation	Biomass (1.14 g/L)	Nitrate (96.99%, 30 d), Phosphate (83.11%, 30 d)	[47]

## Data Availability

The original contributions presented in the study are included in the article, further inquiries can be directed to the corresponding authors.
